# Swelling-based gelation of wet cellulose nanopaper evaluated by single particle tracking

**DOI:** 10.1080/14686996.2022.2153622

**Published:** 2023-01-03

**Authors:** Shohei Moriwaki, Itsuo Hanasaki

**Affiliations:** Institute of Engineering, Tokyo University of Agriculture and Technology, Tokyo, Japan

**Keywords:** Cellulose nanofiber, nanopaper, gel, hydrogel, swelling, single particle tracking, soft matter, microscopy, rheology, gelation

## Abstract

Nanopapers fabricated from cellulose nanofibers (CNFs) drastically swell to form hydrogels when they are in contact with water. This gelation makes contrast with conventional papers that simply deform without drastic volume increase. While the volume increase is qualitatively obvious from the macroscopic visual inspection, its quantitative understanding is nontrivial because of the difficulty in the detection of the boundary between the nanopaper hydrogel and the residual or extra water. In this study, we use single particle tracking (SPT) to reveal the swelling-based gelation phenomenon of cellulose nanopapers. The diffusive dynamics of probe particles uncovers the transient process of swelling, and equilibrium analysis reveals the dependence of volume increase fundamentally dependent on the amount of water to be in contact with the nanopapers. Comparison with the aqueous CNF dispersion without drying reveals the difference in the texture of the nanopaper hydrogels from them.

## GRAPHICAL ABSTRACT

**Figure:**
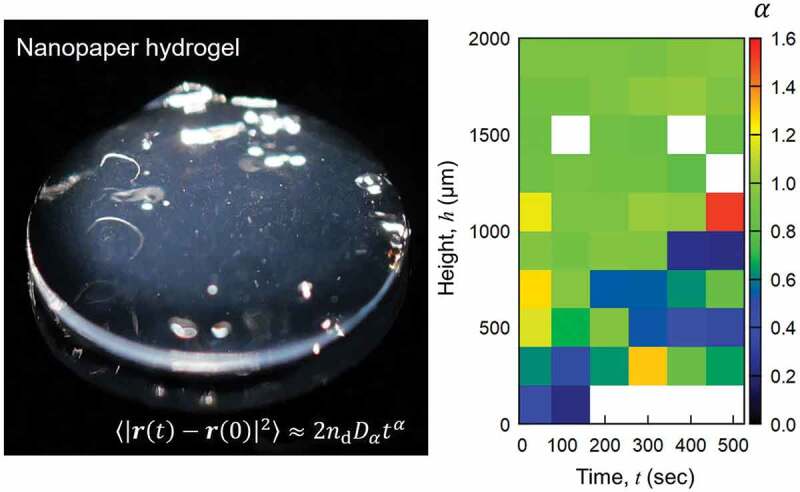

## 1. Introduction

Physical characteristics of gels are typically controlled by the concentration of dispersed filaments and particles when component materials and thermodynamic conditions are fixed. Nevertheless, there are nonequilibrium factors that can affect them. The initial concentration can vary the rheological path in the drying process from sol to gel, which has been revealed in the fabrication of cellulose nanopapers [1]. Cellulose nanopapers [2–5] are sheet-shaped material made of cellulose nanofibers (CNFs) [6], and fabricated by drying the suspension of CNFs in water. While it is rather difficult to concentrate CNFs beyond a few percent in a hydrogel state, drying up CNF suspension results in the thin film structure of nanopapers. The fine texture realized by CNFs with nanometer widths makes nanopapers transparent in contrast to the conventional papers [7]. This fine texture is also recognized as advantageous for printed electronics [8] where conductive nanoparticles are desired to be kept on the surface while the nanopaper is easily miscible with dispersant water. Besides plenty of demonstrations in the printed electronics [8], stimuli responsive functionality based on humidity is explored as well [9]. The actuation by humidity or wetting is a direct consequence of deformation by water [10].

In fact, it is easily confirmed that cellulose nanopaper drastically swells by soaking liquid water. The swelling behavior of nanopaper is regarded as gelation when the amount of water is sufficient, and it has recently been shown to be useful for coatings on Cu electrodes for cohesive protection against water-induced short-circuit failures [11]. Although it is well-known that paper-like material deforms substantially by soaking water, it is not usually regarded as a gel. In this sense, gelation by swelling of wet nanopaper is characteristic to CNFs. In general, the possible applications of CNF-based hydrogels are not limited to the insulation, but includes load-bearing situations [12,13] and wound healing [14]. Based on the environmental friendliness, the applications include soil conditioning [15]. The time dependence of the phenomena can be useful for drug delivery system at microscopic scales [16]. The fabrication process and material characterization of composite combined with CNFs [17–19] need the basic understanding of swelling. The substantial state transition of nanopaper in contact with water is also important for their applications as micro paper-based analytical devices (*µ*PADs) [20].

However, the elementary process or basic mechanism of this gelation by swelling of nanopaper has not been fully addressed so far. The swelling of TEMPO-oxidized nanocellulose [21] has been studied with pulp fibers combined with CNFs instead of nanopapers. There are reports to characterize various forms of cellulose gels [22] and gel characteristics of CNF paying attention to pH and salt concentrations [23], but how it is formed or swelling behavior is beyond the scope of such existing studies. Besides the reports on CNFs, a recent report of ‘swelling-induced gelation’ [24] proposes the gel formation from the sol while the location is the swelling substrate of covalently cross-linked poly(acrylamide) (PAAm). The clear volume evaluation in liquid is realized by optical microscopy observation with a spherical sample. This approach is based on the isotropic nature of the sample. On the other hand, CNFs are reported to align by various perturbation [25]. The isotropy or spatial symmetry of the texture is expected to be modified in the drying process [26]. Therefore, it is also essential to focus on the sheet structure of nanopaper as the initial condition of swelling. In addition, it is nontrivial to prepare spherical solid sample made of CNFs alone.

Thus, in spite of the drastic phenomena qualitatively obvious from visual inspection, there are various missing information on the swelling-based gelation of nanopapers. For example, if the nanopaper is in contact with insufficient amount of water, it might not be the maximum swollen volume. On the other hand, it is nontrivial to determine the maximum volume of swollen nanopaper in liquid water because of the unclear boundary between the gel made of nanopaper and liquid water. Easily available volume fraction of CNF is a few percent at maximum today, and gelation takes place from lower concentration [27–30]. Therefore, the free volume fraction of CNF hydrogels formed from nanopaper in contact with sufficient liquid water is unknown. Furthermore, it is not clear hydrogel from swollen nanopaper is to what extent different from those without complete drying. Since the swelling of gels generally experience the nonuniform process because liquid is supplied only from the interface [31], swelling from dry nanopaper instead of aerogel might keep the nonuniformity after the completion of swelling. This viewpoint calls for microscopic understanding of structural characteristics in the wet state, where scanning electron microscope (SEM) does not have access.

Therefore, we focus on this nanopaper gelation by microrheological approach based on the single particle tracking (SPT) of optical microscopy [29]. SPT-based characterization of aqueous CNF dispersion is effective not only in equilibrium states [30] but also during the nonequilibrium process of drying [1,32]. In this study, we employ this approach to evaluate the swelling behavior of nanopapers. In particular, we investigate the microrheology of cellulose nanopaper hydrogels with different original thicknesses of nanopapers in the transient process of swelling and in the equilibrium state. The generalized diffusion coefficient and its scaling exponent for different position in the thickness direction reveal the transient non-uniformity in the swelling process. The comparison with aqueous CNF dispersion without drying reveals the difference of textures.

## 2. Methods

### 2.1. Fabrication of cellulose nanopaper

**Figure 1.**
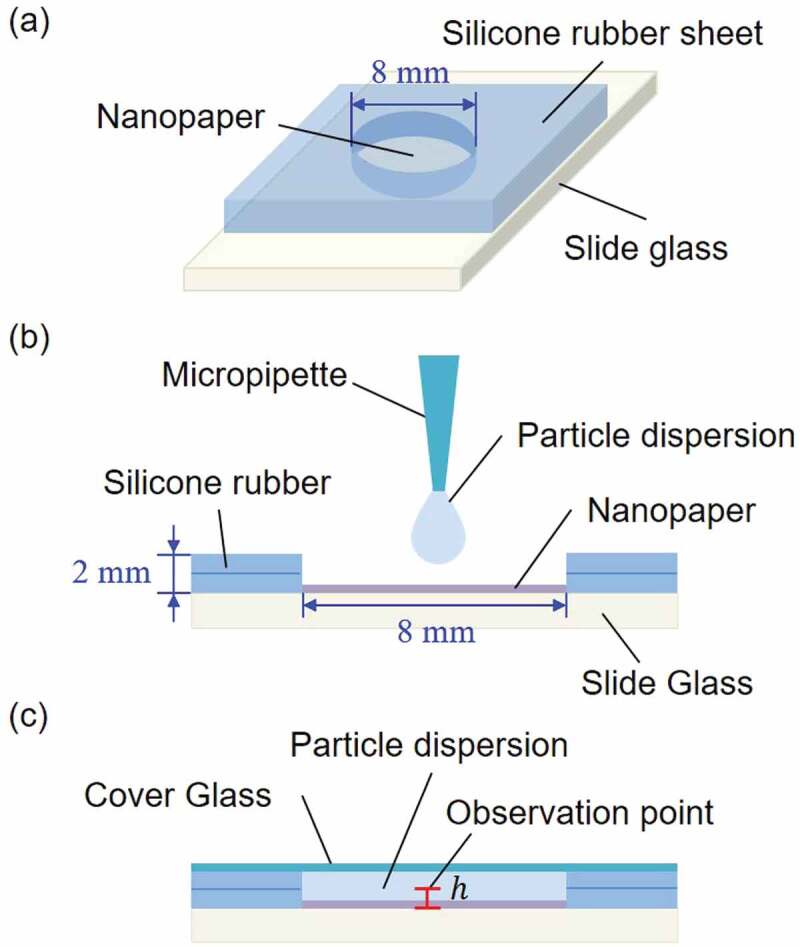
Schematic diagram of the hydrogel of CNFs by swelling of nanopaper: (a) circular disk nanopaper is prepared in a container with a diameter of 8 mm, and (b) water is added to the height of ca. 2 mm, then (c) the container is sealed by cover glass on top of the container.

First, the nanopaper was prepared on a substrate surface. The substrate was made of slide glass (S9915, Matsunami Glass Ind., Ltd., Japan), and the nanopaper was fabricated by drying the aqueous solution of CNFs with the boundary condition of cylindrical side wall made of silicone (SR-50, TIGERS POLYMER CORPORATION) as shown in Figure 1(a). The aqueous solution of CNFs was prepared from TEMPO-oxidized CNFs with an original concentration of 2.2 wt% (I-2SX, DKS Co., Ltd., Japan). The ζ potential of TEMPO-oxidized CNFs is reported to be −75 mV [33]. The microrheological evaluations of the TEMPO-oxidized CNFs in water under equilibrium and nonequilibrium conditions are detailed in Refs [30] and [1], respectively. The 2.2-wt% CNFs was diluted by purified water (Purified water, KENEI Pharmaceutical Co., Ltd., Japan). We prepared different thicknesses of nanopapers by variation of CNF mass $m_{CNF}$ per unit bottom area as summarized in Table 1. We also prepared two types of samples for each $m_{CNF}$. In addition to the simple pure nanopaper adhered to the glass surface, nanopaper embedded with probe particles was prepared for the SPT analysis except for the thinnest nanopaper sample. The probe polystyrene particles had a diameter of 1 µm (Micromer: 01-00-103, micromod Partikeltechnologie GmbH, Germany). The concentration of particles were 8$\times 10^{-3}$ wt% in the aqueous dispersion of CNFs. In all cases, the dilution and mixing of the sample was conducted using the mixer (AR-100, THINKY CORPORATION, Japan) and ultrasonic homogenizer (NR-300MH12 with NR-300MT12, Microtec Co., Ltd., Japan) for the samples in the vial containers (1392-150-SS-LP-CS, FUKAEKASEI Co.,LTD and WATSON Co., Ltd., Japan). The mixer was operated at 2000 rpm of revolution with 800 rpm of rotation for 5 min. Then, ultrasonic homogenizer was operated at PWM 30% of power for 2 min. Finally, degassing operation of the mixer was conducted by 2200 rpm of revolution for 5 min. The sample liquid was dried at 40${\;}^{\circ }$C in a chamber (THR030FA, Advantec co., ltd., Japan) without humidity control for at least 24 hours. The pipetting precision for dilution was supported by the measurement of the mass by electronic balance (MS205DU, Mettler-Toledo, LLC, Switzerland).

**Table 1.** Fabrication conditions of nanopapers: initial mass concentrations $C_{CNF}$ and $C_{p}$ of CNFs or CNF⋅particle dispersion, volume $V_{sol}$ of the aqueous dispersion, and CNF mass $m_{CNF}$ per unit bottom area.

Label	$C_{CNF}$ (wt%)	$C_{p}$ (wt%)	$V_{sol}$ (μL)	$m_{CNF}$ (kg/m${\;}^{2}$)
1	0.05	0	20.0	1.99 $\times 10^{-4}$
2	0.05	0	177.3	1.76 $\times 10^{-3}$
3	0.05	8.0 $\times 10^{-3}$	177.3	1.76 $\times 10^{-3}$
4	0.10	0	177.3	3.53 $\times 10^{-3}$
5	0.10	8.0 $\times 10^{-3}$	177.3	3.53 $\times 10^{-3}$
6	0.15	0	177.3	5.29 $\times 10^{-3}$
7	0.15	8.0 $\times 10^{-3}$	177.3	5.29 $\times 10^{-3}$
8	0.18	0	177.3	6.35 $\times 10^{-3}$
9	0.18	8.0 $\times 10^{-3}$	177.3	6.35 $\times 10^{-3}$

### 2.2. Measurement of dry nanopaper thickness and surface topography

The thicknesses of fabricated nanopapers adhered on slide glass surface were measured by optical interference apparatus (F20, Filmetrics Japan, Inc.). The mode of analysis was based on the Fourier transform. The settings of refraction indices were Air, n=1.46, BSG from the top to the bottom, respectively. The initial values of film thickness before convergence were defined as 5±5μm. The validation of measurement results was based on the goodness of fit (GOF) being more than 0.8. Horizontal centers of ten film samples were employed for the measurements of each fabrication condition, and the mean values were defined as the representative film thickness. The measurement results are summarized in Table 2. Hereafter for clarity, we employ the measurement results of thickness for cases 2, 4, 6, and 8 also as the sample labels of 3, 5, 7, and 9, respectively.

**Table 2.** Thickness $L_{t}$ of each nanopaper. The label numbers correspond to those in Table 1.

Label	$C_{CNF}$ (wt%)	$V_{sol}$ (μL)	$L_{t}$ (µm)
1	0.05	20.0	0.15
2	0.05	177.3	0.45
4	0.10	177.3	0.97
6	0.15	177.3	1.68
8	0.18	177.3	2.12

**Figure 2.**
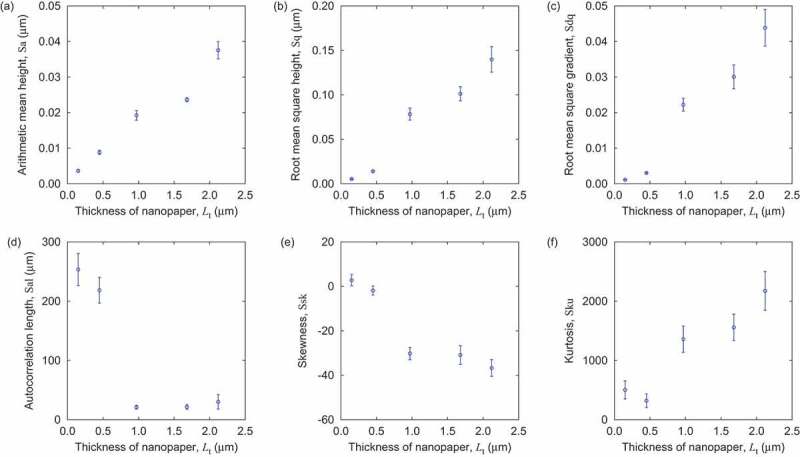
Surface characteristics of cellulose nanopaper samples as a function of thickness. The error bars indicate the standard errors.

We have also evaluated the surface topography by surface roughness using the optical measurement system (Profilm3D, Filmetrics Japan, Inc.). We prepared ten nanopaper samples for each thickness condition, and measured a central area of 1.93 ×1.68 $mm^{2}$ for each sample. After horizontal correction, we evaluated the surface roughness criteria based on ISO 25178 using the software utility of the measurement system. The obtained results are summarized in Figure 2. Both arithmetic height and root mean square height increase with nanopaper thickness $L_{t}$. Since root mean square gradient also increases with $L_{t}$, the area roughness increases with $L_{t}$. On the other hand, the autocorrelation length was insensitive to $L_{t}$ when $L_{t}\geq 1\mu m$. The negative values of skewness suggests that there are more fine texture of concave parts rather than convex parts. The increase of kurtosis with $L_{t}$ is of a similar nature to that of root mean square gradient.

### 2.3. Optical microscopy movie of nanopaper in contact with water

We monitor the swelling-based gelation of nanopaper by recording the movie of optical microscopy. The sample configuration is shown in Figure 1(b,c). The water dispersed with polystyrene particles with a diameter of 1 *µ*m (Micromer: 01-00-103, micromod Partikeltechnologie GmbH, Germany) was dropped on the nanopaper with a volume of 112 *µ*L, and sealed with the cover glass to avoid water evaporation. The particle concentration in this dispersion was 0.025 wt% for the case with label 1 and 0.012 wt% for cases with labels 3, 5, 7, and 9, respectively. Thus, the same products of probe particles were used for both the water droplets and the nanopapers. Whereas case 1 has probe particles only in the water dispersion as the initial condition, cases 3, 5, 7, and 9 contains probe particles both in the nanopaper and water dispersion from the beginning. This is intended for embedding sufficient concentrations of probe particles in the swelling nanopaper also in the transient process.

The movie recordings of optical microscopy were conducted for both transient process and equilibrium state of the swelling-based gelation of nanopapers. The horizontal position was always defined as the center of the container. The vertical positions h (cf. Figure 1(c)) were h=100μm and 200-μm spacing from there to have ten points of measurement positions. The influence of refraction index in water was taken into account to determine the actual values of h. More specifically, the *apparent* distance between the lower surface of the cover glass to seal the container and the upper surface of the slide glass was determined, where the latter was defined by the focal position of particles adhered to the slide glass. Since the *actual* distance had been confirmed by the measured thickness of the silicone sheet, the ratio of actual to apparent heights of the sample domain between the glass surfaces was determined before the movie capture experiments. An sCMOS camera (Zyla5.5, Andor Technology Ltd., UK) was used at a frame rate of 100 fps with an exposure time of $9.0\times 10^{-4}$ s with an image size of 512 ×512 $px^{2}$. This image size corresponds to the square of 83 *µ*m on a side by 6.5 *µ*m/px of the camera pixel pitch and the objective lens of 40× mounted on the inverted optical microscope (IX73, Olympus Corporation, Japan) with phase contrast setting. A single movie data consisted of $10^{3}$ frames, where each frame is a grayscale 16-bit TIFF image.

In the measurements of transient swelling process, the recording started from 30 s after dropping the particle dispersion of water onto a dry nanopaper sample, and the 10 s of movie capture was repeated every 90 s of intervals. The measurements at ten points of h for the transient process were enabled by the samples of ten nanopapers. A single experiment of transient process consisted of time series movie capture under the condition of a single value of h for a single nanopaper sample. The movie recording for equilibrium SPT analysis was conducted in time series as summarized in Table 3. Three times of measurements at each height h at different time point after first incubation time of 30 min are intended not only for the sufficient amount of data but also for the examination of equilibrium. The specific values of timing do not make significant difference as far as the equilibrium condition is satisfied. In summary, a single nanopaper sample was used for ten points of h in the equilibrium movie capture experiments, whereas ten nanopaper samples were used for the movie capture of transient process at ten h.

**Table 3.** The movie capture procedure for the equilibrium SPT analysis indicated by the heights h (cf. Figure 1) as a function of time point *t* to start each recording. Three movies were recorded for each h.

Measurement height	Time *t* (min.)		
*h* (μm)	1st	2nd	3rd
1900	48	78	108
1700	46	76	106
1500	44	74	104
1300	42	72	102
1100	40	70	100
900	38	68	98
700	36	66	96
500	34	64	94
300	32	62	92
100	30	60	90

### 2.4. Data analysis based on SPT

We extracted the numerical data of particle trajectories from the movie data using the algorithm of Sbalzarini and Koumoutsakos [34]. The input parameters for the analysis were decided as follows: Particle radius 6 px, Intensity percentile 0.1 %, Cutoff score 0, Link range 1, and Displacement threshold 2 px, respectively. In this study, we employ the framework of generalized diffusion [35,36] to evaluate the probe dynamics. Namely, the time evolution of the mean squared displacement (MSD) is expressed as:

(1) $$\langle |r(t)-r(0){|}^{2}\rangle \approx 2n_{d}D_{\alpha }t^{\alpha },$$

where $\left\langle |r(t)-r(0){|}^{2}\right\rangle$ indicates the MSD by ensemble average ⟨⟩ of squared displacements $|r(t)-r(0){|}^{2}$ based on the particle position r(t) at time t related to arbitrary t=0 with an observation dimension $n_{d}$. The set of generalized diffusion coefficient $D_{\alpha }$ and its scaling exponent α indicate the characteristics of the surrounding media. In particular, α=1 corresponds to the normal diffusion and α<1 indicates the subdiffusion. This evaluation is useful for the characterization of the media surrounding the probe particles [1,29,30,32]. In the numerical evaluation, the ensemble average was calculated by the following definition:

(2) $$\frac{\sum_{i=1}^{N_{I}}\sum_{j=1}^{N_{Fi}-N_{itvl}}|r_{i}(t_{j+N_{itvl}})-r_{i}(t_{j}{)|}^{2}}{2n_{d}\sum_{i=1}^{N_{I}}\left(N_{Fi}-N_{itvl}\right)}\approx D_{FB\alpha }\Delta t_{spn}^{\alpha_{FB}},$$

where $N_{I}$ and $N_{Fi}$ are the number of particles detected and the number of consecutive frames for i-th particle, respectively. $\Delta t_{spn}\equiv t_{j+N_{itvl}}-t_{j}$ is the time span for the evaluation of MSD. $N_{itvl}=\Delta t_{spn}/\Delta t_{frm}$ is the number of frames corresponding to the time span, where $\Delta t_{frm}$ is the frame interval $10^{-2}$ s of the camera recording. In this definition, each frame is weighted as equivalent, which we call frame-based (FB) averaging, in contrast to the individual-based averaging [29]. The numerical evaluation of $D_{FB\alpha }$ and $\alpha_{FB}$ is completed by the fitting of obtained results to the model equation. The fitting procedure was based on the linear fitting for the logarithm of the power law equation, except for that some cases with effectively fixed trajectory required explicit nonlinear fitting of power law equation including the origin of (MSD, t)=(0,0) to define the non-negative scaling exponent for the numerical fitting. Since the time evolution of the MSD is an important characteristic, we also examine the distribution of MSD per individual sample (IS) of particle i:

(3) $$\langle \Delta r^{2}(\Delta t_{spn})\rangle_{ISi}=\frac{\sum_{j=1}^{N_{Fi}-N_{itvl}}|r_{i}(t_{j+N_{itvl}})-r_{i}(t_{j}{)|}^{2}}{N_{Fi}-N_{itvl}}.$$

The distribution of the logarithm of this quantity reveals the multimodal diffusion when significant [30,37].

## 3. Results and discussion

**Figure 3.**
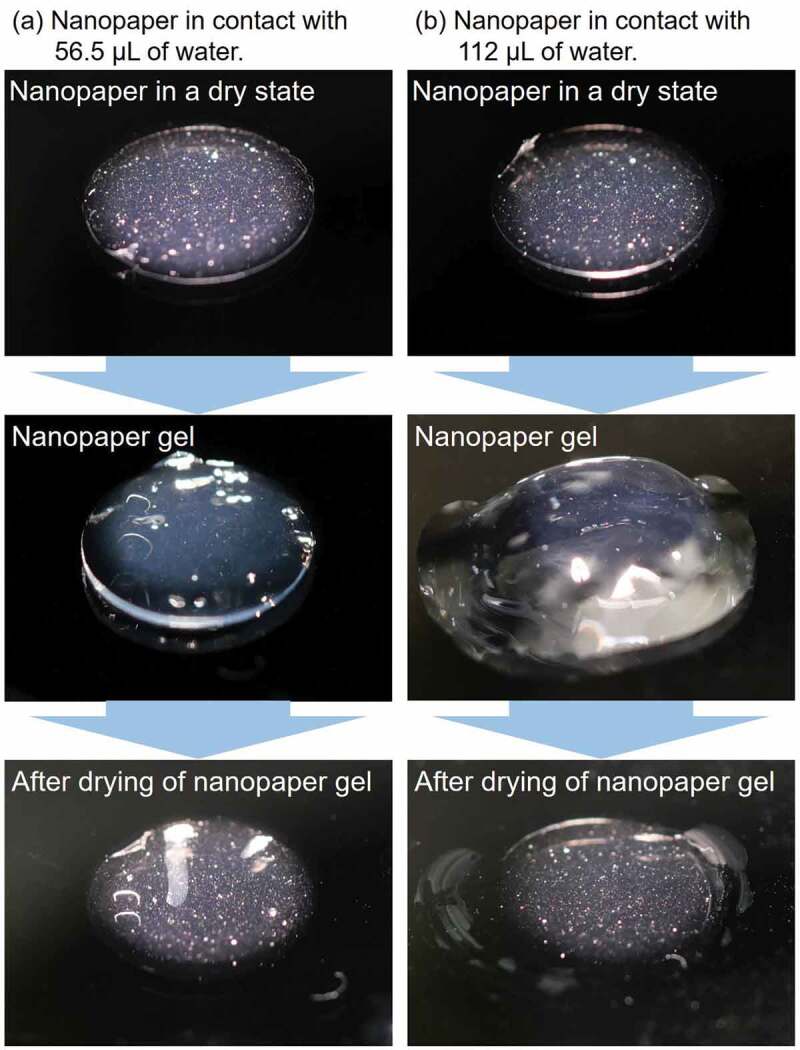
Digital camera images of the swelling of wet nanopaper. The top panels show the original state of the nanopaper fabricated from 6.35$\times 10^{-3}kg/m^{2}$ (cf. Table 1) CNF in a cylindrical container well with a bottom diameter of 8 mm (cf. Figure 1) without silicone sidewall before addition of water. The nanopapers swell in contact with different amounts of water to form hydrogels with different volume as shown in the middle panel. The sidewall made of silicone sheet (cf. Figure 1) was removed after addition of water before photographing the hydrogel. Drying of the hydrogel results in the different macroscopic shapes as shown in the bottom panel.

Figure 3 shows the macroscopic observation of swelling-based nanopaper gelation. When a nanopaper gets in contact with liquid water, the swelling behavior is observed. The specific condition of nanopaper fabrication in Figure 3 corresponds to Label 8 in Tables 1 and 2. The difference in the amount of water added to the nanopaper appears to result in the different volumes of nanopaper hydrogels. If a nanopaper with a specific thickness has an intrinsic gel volume, a sufficient amount of water would cause the formation of subdomains of gel and liquid water. However, if a nanopaper does not have a specific intrinsic gel volume to be formed, the variation of the amount of water to be in contact would not generate such a domain boundary. Figure 3 also shows the shape of samples after drying of the swollen nanopaper hydrogels. Whereas Figure 3(a) shows similar appearance of macroscopic shape to the original dry nanopaper, Figure 3(b) shows the clear difference in the shape compared to the original state of the nanopaper. In other words, the nanopaper could not sustain the hydrogen bond network to restore the original shape. It is nontrivial to define what the intrinsic volume of the gel when the structural rigidity is not sufficiently kept.

**Figure 4.**
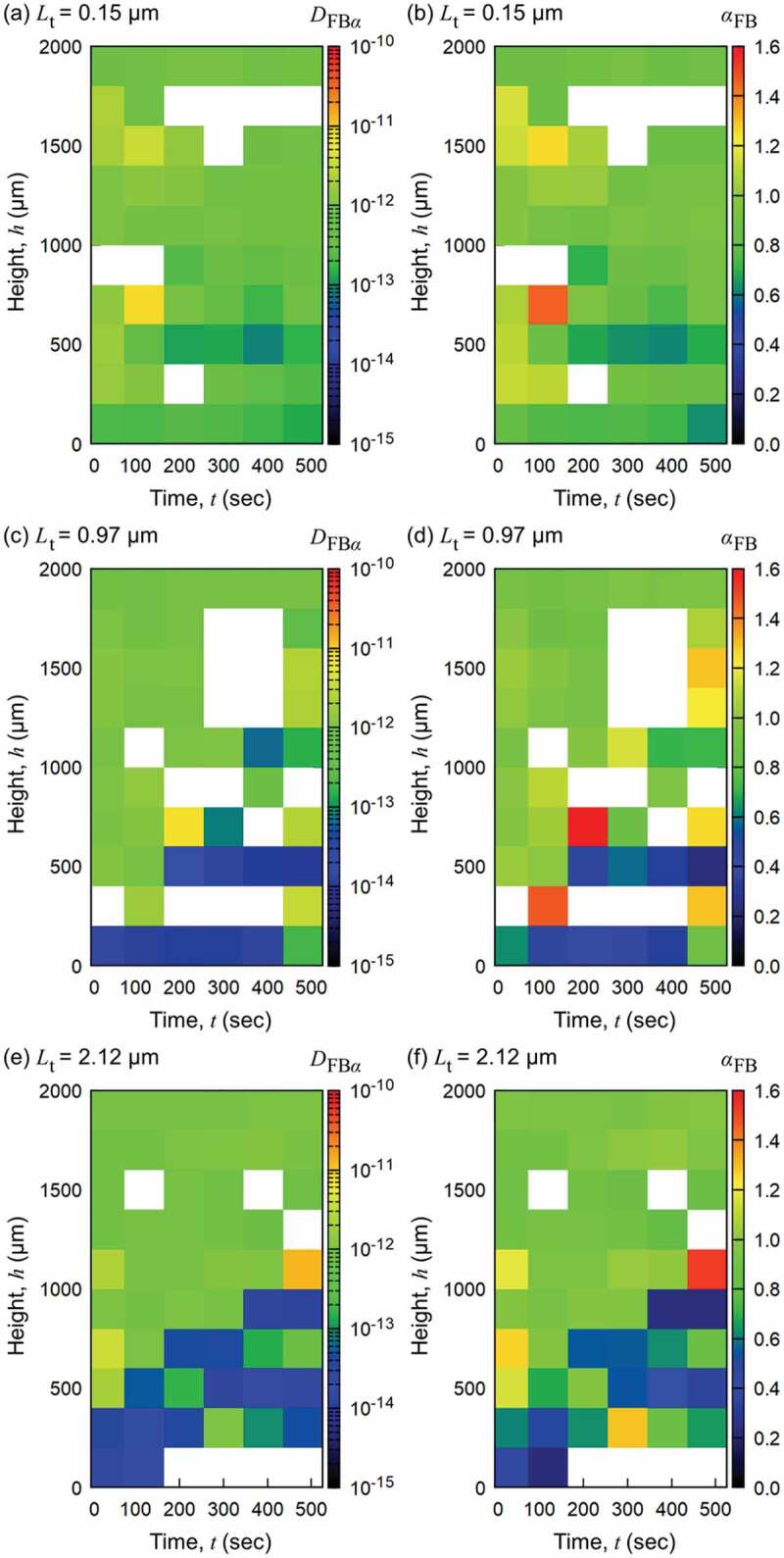
Time evolution of generalized diffusion behavior of probe particles in the swelling hydrogels of nanopapers with original thicknesses of (a), (b) 0.15 *µ*m, (c), (d) 0.97 *µ*m, and (e), (f) 2.12 *µ*m, respectively. (a), (c) and (e) indicate the time evolution of $D_{FB\alpha }$, and (b), (d) and (f) indicate that of $\alpha_{FB}$, respectively. Trajectories were not obtained for the blank (white) grids (squares) mainly due to the unclear particle images.

Therefore, we examine and analyze the existence of CNFs at specific positions in the cylindrical well (cf. Figure 1) through the Brownian motion of probe particles. Figure 4 shows the time evolution of generalized diffusion coefficient $D_{FB\alpha }$ and its exponent $\alpha_{FB}$ of probe particles for different original thicknesses of nanopapers. Only the transient behaviors within 500 s are analyzed. Both $D_{FB\alpha }$ and $\alpha_{FB}$ indicate the similar trend for each sample. The diffusive behaviors of probe particles in the swelling nanopapers significantly slows down compared to the outside of them, and it is basically subdiffusive. However, occasional detection of $\alpha_{FB}>1$ is also recognized for all of the three samples with different original nanopaper thicknesses. The significant subdiffusive characteristics instead of normal diffusion suggest the network structure of CNFs in the gel state rather than the isolated CNFs in the sol state. The occasional superdiffusive behavior is attributed to the persistent particle dynamics driven by the swelling motion of CNF network. Gradual increase of domain with slower and confined diffusion of probe particles indicates the transient swelling behavior of nanopaper. Although the case of $L_{t}=0.15\;\mu m$ is not clear, the cases of $L_{t}=0.97\;\mu m$ and 2.12 *µ*m indicates the 2 to 3 *µ*m/s in the nanopaper front propagation. The unclear dependence of swelling speed on the original thickness is partly attributed to the non-uniformity of swelling dynamics.

**Figure 5.**
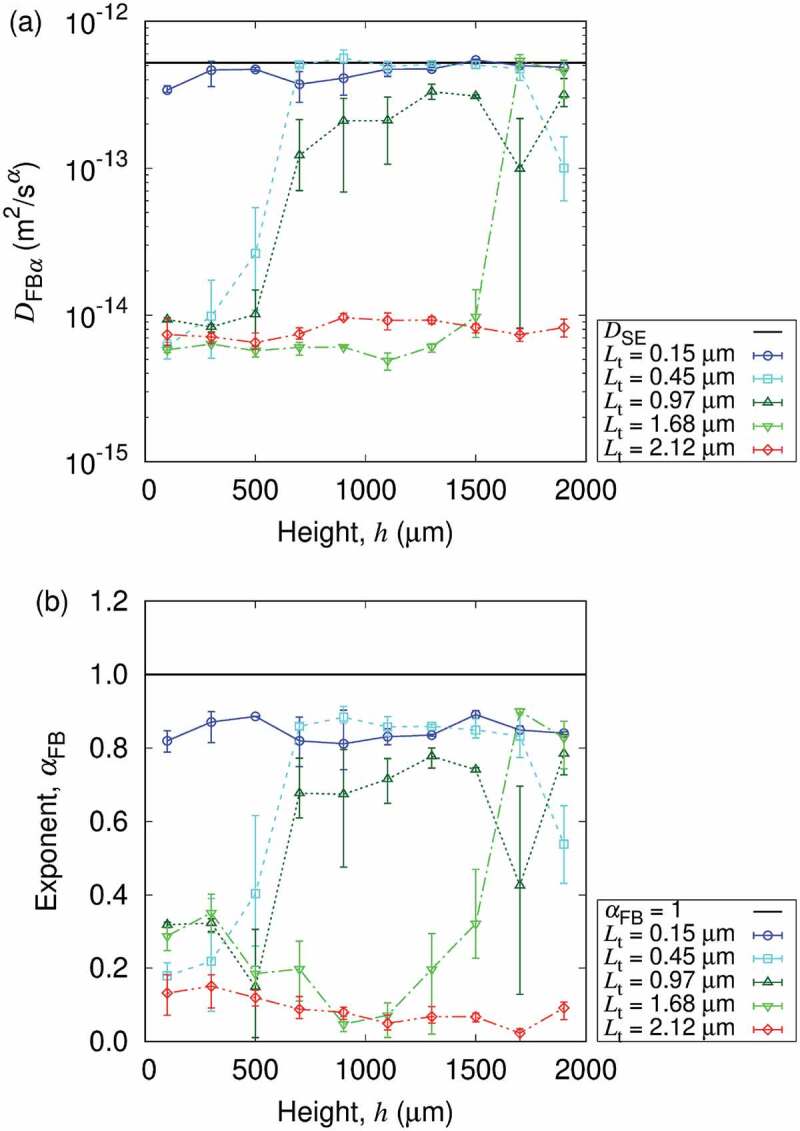
Height h dependence of (a) generalized diffusion coefficient and (b) its scaling exponent for different original thicknesses $L_{t}$ of nanopapers in equilibrium. h=0 corresponds to the bottom wall position (cf. Figure 1). The error bars indicate the minimum and maximum values obtained from three time points with 30-min intervals. The dilute particle diffusion without CNFs predicted by the Stokes-Einstein relation is also shown for reference.

The transient swelling behavior of nanopaper is observed for at least 5 min from the contact event with water, and the time resolution of 1.5 min was sufficient for the detection of swelling. Based on this observation, we evaluated the equilibrium states of swollen nanopaper hydrogel. Figure 5 shows the height dependence of the generalized diffusion coefficient and its exponent as a function of position h in the thickness direction. The equilibration is confirmed by the evaluation at three time points with 30-min intervals. The thin nanopaper with originally ca. 0.15μm of thickness shows almost the same $D_{FB}$ with slight yet significant subdiffusive characteristics, and the sample was uniform in height. On the other hand, the thick nanopaper with originally ca. 2.12μm of thickness shows significantly lower $D_{FB}$ with $\alpha_{FB}\ll 1$. Nevertheless, the sample was uniform in height, which is similar to the thinnest nanopaper sample. Thus, the nanopaper with different thicknesses in dry state *can* reach the different concentration of CNFs uniformly distributed in height, when in contact with the same amount of water confined in the same boundary condition defined by the container. This result can be either interpreted as (1) the cellulose nanopaper does not have intrinsic gel volume but the volume depends on the water amount to be in contact, or (2) the amount of water in contact was still insufficient to reveal the intrinsic volume. However, the swollen nanopaper with this combination of nanopaper thickness and liquid volume (cf. Figure 3(b)) did not sustain its macroscopic shape against its gravity (on earth). Therefore, no intrinsic gel volume is the plausible interpretation. It is also reasonable from the fact that we had detected the partial structural order of CNFs in the aqueous dispersion of CNFs without drying in our previous study [30].

Nevertheless, looking into more samples with further variation of nanopaper thickness, we also find the coexisting characteristics. The equilibrium state of swollen nanopapers can also exhibit nonuniformity in the local concentration or structures of CNFs as revealed by this SPT analysis. Figure 5 shows the two peaks in the distribution of both $D_{FB\alpha }$ and $\alpha_{FB}$. In between, the distribution is sparse and the evaluation result shows large scattering. Therefore, when dry cellulose nanopapers swell with water, the probe particles with a diameter of 1µm experience the confinement of $\alpha_{FB}∼0.1$ and $D_{FB\alpha }\text{\textbackslash{}\textasciitilde{}}10^{-14}m^{2}/s^{\alpha }$ but further swelling can lead to more dilute network of typically $\alpha_{FB}∼0.7-0.9$ and $D_{FB\alpha }\text{\textbackslash{}\textasciitilde{}}D_{SE}>10^{-13}m^{2}/s^{\alpha }$. This nonuniformity is regarded as equilibrium from the view point of time scale of $10^{2}$ min. (cf. Table 3). The two peaks of texture characteristics are interpreted as the existence of high CNF concentration domain where insufficient swelling is induced by the contact with water. Since the high concentration domains were observed in the bottom side of the sample, the binding of CNFs to the bottom wall of the container is likely to have contributed to the remaining high CNF concentration domain.

**Figure 6.**
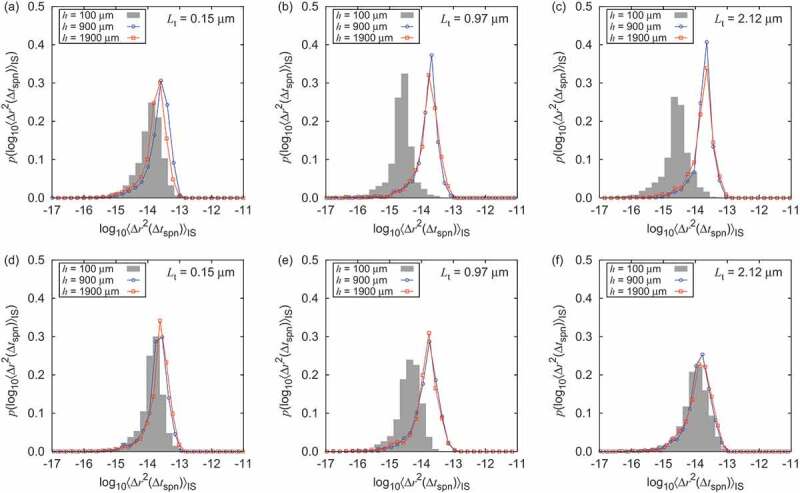
Distribution of the logarithm of squared displacement at different height h position of hydrogel swollen from nanopapers in the transient state t=30 s ((a), (b), and (c)) and in the third recording of the equilibrium state ((d), (e), and (f)) with original thicknesses of (a), (d) 0.15 *µ*m, (b), (e) 0.97 *µ*m, and (c), (e) 2.12 *µ*m for $\Delta t_{spn}=10^{-2}$ s, respectively.

**Figure 7.**
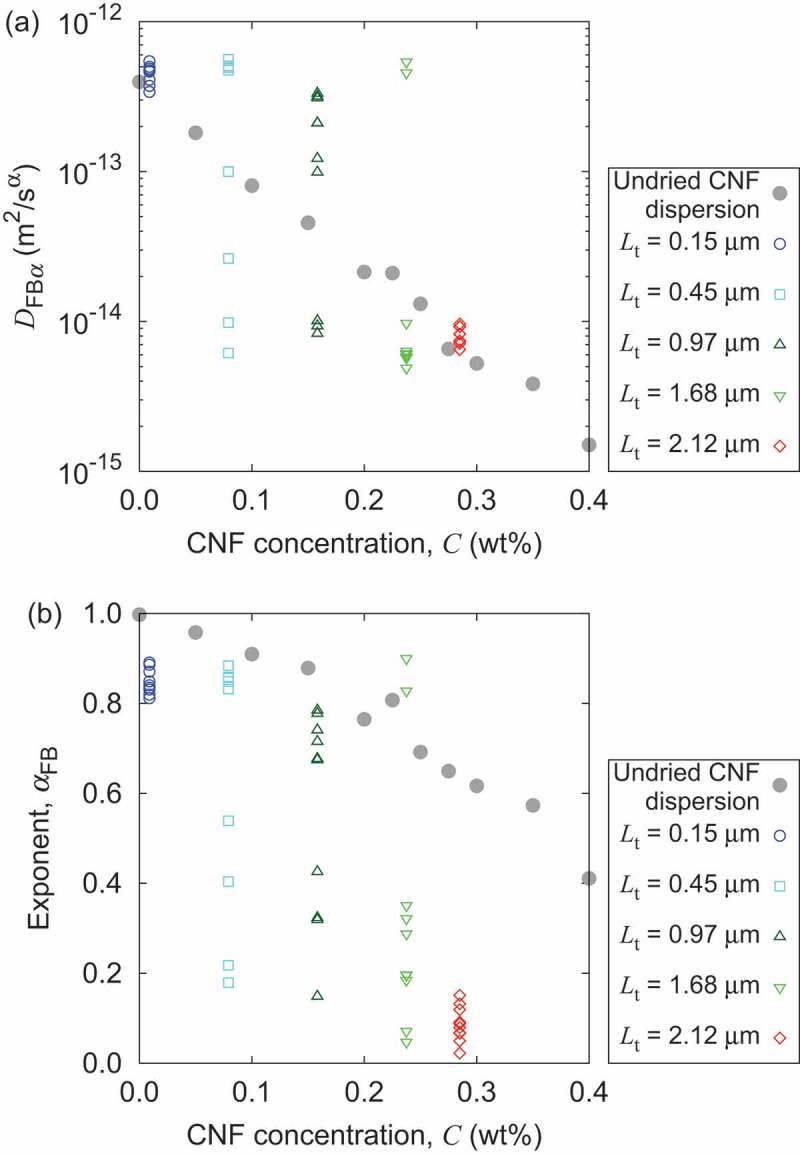
Comparison between nanopaper hydrogel with aqueous CNF dispersion through the generalized diffusion behavior of 1 *µ*m probe particles. The quantities of the undried CNF dispersion is cited from Ref. [30], where only the mean values are defined. CNF concentration C of nanopaper hydrogels are nominal values defined by the mass of CNFs per bottom area divided by added water amount in the cylindrical container. Ten points per single value of C correspond to the ten h position in Figure 5 for a single original nanopaper thickness $L_{t}$.

The ratio of the number of probe particles in the dried nanopaper before hydrogel formation to that in the water to be added for swelling nanopaper is 1.06. Therefore, the diffusive dynamics would be multimodal in the overall sample if the probe particles embedded in the nanopaper often bound to the CNFs due to the drying. The multimodality is easily found by the distribution of logarithm of the squared displacements [30,37]. However, Figure 6 shows that the diffusive behavior is unimodal, in spite of highly nonuniform profile of $\alpha_{FB}$ observed in Figures 5 and 7. Figure 6 indicates that the characteristic space scale of nonuniformity is typically beyond $10^{2}\mu m$ corresponding to the image size of the microscopy movie. The sample with $L_{t}=0.97\mu m$ shows different range of displacement distribution depending on h while keeping it unimodal. This scale of texture nonuniformity (Figure 6(c)) in the transient period for the sample of $L_{t}=2.12\mu m$, disappeared after equilibration (Figure 6(f)).

When the profile of generalized diffusion coefficient and its scaling exponent is uniform, the uniformity of the nanopaper gel can provide the overall concentration of the CNFs in water by mass of CNFs and water amount. Then, the comparison to the aqueous dispersion of CNFs without drying [30] is valuable for the understanding of possible texture variation due to the experience of drying. Figure 7 shows the comparison of $D_{FB\alpha }$ and $\alpha_{FB}$ of 1-µm probe particles in water with CNFs with and without the drying. The comparison is based on the same (nominal) overall CNF concentration. The originally thin nanopaper ($L_{t}=0.15\;\mu m$) shows similar $D_{FB\alpha }$ and $\alpha_{FB}$ to those of the CNFs in water without drying, when swollen by addition of water. The mean of $D_{FB\alpha }$ is similar for each (nominal) concentration CNF. On the other hand, the $\alpha_{FB}$ of swollen hydrogel from nanopapers are smaller than that of aqueous dispersions of CNFs without drying, and this deviation is more pronounced when the original thickness $L_{t}$ of nanopaper is larger. This decrease of $\alpha_{FB}$ by drying is attributed to the difference of CNF network structures between them. Since nanopaper fabrication leads to dense network structures of CNFs with hydrogen bonding, the swollen hydrogel might have higher concentrations of hydrogen bonds compared to those of aqueous dispersion of CNFs without drying. In other words, once the dense hydrogen bond networks of CNFs are formed by complete drying (to the ambient humidity), swelling to the nominal concentration of CNFs to less than 0.3 wt% may still keep significantly larger number density of hydrogen bonds without sufficient dissociation to the level of the aqueous CNF dispersion with the same nominal CNF concentration without drying (possibly produced by concentration from more dilute dispersion). If that is the case, the lower $\alpha_{FB}$ is a consequence of finer network that more often hinders the diffusive motion of probe particles.

## 4. Conclusions

Based on SPT, we have revealed the characteristics of swelling-based gelation process that occurs when cellulose nanopapers get in contact with water. We prepared different thicknesses of nanopapers and added plenty of amount of liquid water, to the extent that the nanopaper hydrogel cannot sustain the structural order to restore the original shape of nanopaper when dried up. Since the initial condition of nanopapers consists of dense hydrogen bond network that does not allow the spontaneous penetration of probe particles, we embedded the probe particles in the nanopapers to be used for the swelling experiments. We also included the same amount of probe particles in the liquid droplet to be added to the nanopaper. The SPT analysis with different positions in the nanopaper thickness directions at different time points revealed the swelling process roughly at the rate of 2 to 3 *µ*m/s. This is hard to determine by visual inspection when the transparent nanopaper without color is soaked in water. The transient swelling process occasionally induced temporary flow of the media, where probe particles were persistently driven instead of normal diffusion.

The texture uniformity/nonuniformity of the consequent nanopaper hydrogels depended on samples. Both thin and thick nanopapers resulted in the uniform texture examined by the generalized diffusion and its scaling exponent of the probe particles. The simple cellulose nanopapers consisting of TEMPO-oxidized CNFs do not have intrinsic gel volume but the volume depends on the amount of water to be added. On the other hand, some samples left substantial nonuniformity at least within the time scale of $10^{2}$ minutes. The characteristic length scale of nonuniformity was beyond $10^{2}\;\mu m$, as examined by the distribution of the logarithm of squared displacements. The generalized diffusion coefficients $D_{FB\alpha }$ in the nanopaper hydrogels were scattered around those in the aqueous CNF dispersion without drying when compared by the nominal CNF concentrations. The scaling exponents $\alpha_{FB}$ in the nanopaper hydrogels were lower than those without drying. This difference suggests the finer network of CNFs in the hydrogels even when they cannot sustain the structural order against the gravity.

Since the characteristics of confined probe diffusion are a function of nominal CNF concentration, the swelling behavior can be controlled by the available volume for swelling. For example, this can be used for the design of filters and channels in *µ*PADs, where rigid room without swelling works as the boundary conditions. The rate of swelling and apparently unlimited final volume are useful for the design of not only filtration but also drug delivery and cell culturing. The unlimited volume also suggests the importance of water exposure in the context of green electronics or degradable devices, or any applications of cellulose nanopapers for field applications where finite time usage (rather than maximum durable life) is a prerequisite for environmental friendliness.
